# *Mycobacterium avium* subsp. *paratuberculosis* Proteome Changes Profoundly in Milk

**DOI:** 10.3390/metabo11080549

**Published:** 2021-08-20

**Authors:** Kristina J. H. Kleinwort, Bernhard F. Hobmaier, Ricarda Mayer, Christina Hölzel, Roxane L. Degroote, Erwin Märtlbauer, Stefanie M. Hauck, Cornelia A. Deeg

**Affiliations:** 1 Chair of Physiology, Department of Veterinary Sciences, LMU Munich, D-82152 Martinsried, Germany; k.kleinwort@lmu.de (K.J.H.K.); bernhard.hobmaier@lgl.bayern.de (B.F.H.); r.degroote@lmu.de (R.L.D.); 2 Chair of Hygiene and Technology of Milk, Department of Veterinary Sciences, LMU Munich, D-85764 Oberschleißheim, Germany; mayer@gna-bio.com (R.M.); choelzel@tierzucht.uni-kiel.de (C.H.); e.maertlbauer@mh.vetmed.uni-muenchen.de (E.M.); 3 Institute of Animal Breeding and Husbandry, Faculty of Agricultural and Nutritional Sciences, CAU Kiel, D-24098 Kiel, Germany; 4 Research Unit Protein Science, Helmholtz Center Munich, German Research Center for Environmental Health, D-80939 Munich, Germany; hauck@helmholtz-muenchen.de

**Keywords:** *Mycobacterium avium* subspecies *paratuberculosis*, dairy product, milk, differential protein expression, food safety, LC–MS/MS, proteomics

## Abstract

*Mycobacterium avium* subspecies *paratuberculosis* (MAP) are detectable viable in milk and other dairy products. The molecular mechanisms allowing the adaptation of MAP in these products are still poorly understood. To obtain information about respective adaptation of MAP in milk, we differentially analyzed the proteomes of MAP cultivated for 48 h in either milk at 37 °C or 4 °C or Middlebrook 7H9 broth as a control. From a total of 2197 MAP proteins identified, 242 proteins were at least fivefold higher in abundance in milk. MAP responded to the nutritional shortage in milk with upregulation of 32% of proteins with function in metabolism and 17% in fatty acid metabolism/synthesis. Additionally, MAP upregulated clusters of 19% proteins with roles in stress responses and immune evasion, 19% in transcription/translation, and 13% in bacterial cell wall synthesis. Dut, MmpL4_1, and RecA were only detected in MAP incubated in milk, pointing to very important roles of these proteins for MAP coping with a stressful environment. Dut is essential and plays an exclusive role for growth, MmpL4_1 for virulence through secretion of specific lipids, and RecA for SOS response of mycobacteria. Further, 35 candidates with stable expression in all conditions were detected, which could serve as targets for detection. Data are available via ProteomeXchange with identifier PXD027444.

## 1. Introduction

*Mycobacterium avium* subsp. *paratuberculosis* (MAP) is the causative agent of paratuberculosis or Johne’s disease, a chronic granulomatous enteritis of cattle and small ruminants [1,2]. Due to its remarkable similarity to the pathology of Crohn’s disease in humans, it has been discussed for a long time as a possible cause of human morbidity, especially chronic inflammatory bowel diseases (IBD) such as Crohn’s disease and ulcerative colitis [3,4,5,6]. Although there is no definitive proof of MAP being involved in the pathogenesis of the human diseases, it is broadly accepted in the scientific community that human exposure to MAP, especially through contaminated milk and milk products, should be reduced [7]. MAP can either be shed directly into milk by infected cows or be introduced via fecal contamination [8,9,10,11]. Viable MAP were found in pasteurized milk, cheese, and even in dried milk products such as infant formula [12,13,14]. This might have been caused by post-processing contamination, but there is also evidence of MAP being able to survive the pasteurization process [15,16,17,18,19]. The mechanisms behind this tenacity, however, are not fully understood and have been vigorously discussed over the last years [8,20]. Theories include clumping, intracellular localization of MAP in milk, and formation of heat-resistant forms such as endospores [16,21,22].

It is common for bacteria to sense environmental changes and react to it with various stress response mechanisms [23]. Milk in particular is a rather hostile environment for bacteria, due to several mechanisms that inhibit bacterial growth such as the iron binding protein lactoferrin, antimicrobial peptides, and different nutrient sources compared to the bacteria’s physiological environment (i.e., lactose instead of glucose) [24,25]. Significant proteome changes after cultivation in milk compared to cultivation in a standard medium were shown for various bacteria, including *Escherichia coli*, *Lactococcus lactis*, and *Streptococcus thermophilus* [24,26,27]. Therefore, it is not unlikely for MAP to also adapt to such altered environmental conditions by changing several of its physiological properties. A good example for this environment-dependent variation are cell wall-deficient forms of MAP that were repeatedly isolated from human intestines [6,28,29,30]. In a previous study, we showed that after incubation of MAP with peripheral blood mononuclear cell (PBMC) of cows with different immunological phenotypes, the exoproteome of MAP changed according to the immunological phenotype of the leukocytes used for coincubation [31]. For milk, altered properties of MAP were shown by an in vitro experiment, in which incubation of MAP in milk led to faster translocation through a monolayer of Madin–Darby bovine kidney epithelial cells (MDBK) cells [32]. In addition to this, MAP cultured in milk had distinctly different gene expression profiles compared to those cultured in broth (different cell wall lipid profile and enhanced expression of cell invasion-associated genes), suggesting milk as a valid transmission route for MAP to calves and humans [33]. However, changes in gene expression do not necessarily always mean equivalent changes in the proteome. Therefore, it is important to validate those findings on a protein level. Understanding how the MAP proteome changes after being incubated in milk may elucidate the role of milk in MAP transmission, shed light to the question of why MAP can survive pasteurization, and may lead to new possible targets for the detection of MAP in milk and milk products. Therefore, in this study, we investigated possible differential proteome changes in MAP after incubation in milk at 37 °C (simulating the situation in the mammary gland) and 4 °C (for tank milk) in comparison to MAP from M7H9 broth at 37 °C.

## 2. Results

### 2.1. Identification of MAP Proteome with Label-Free Mass Spectrometry

**Figure 1:**
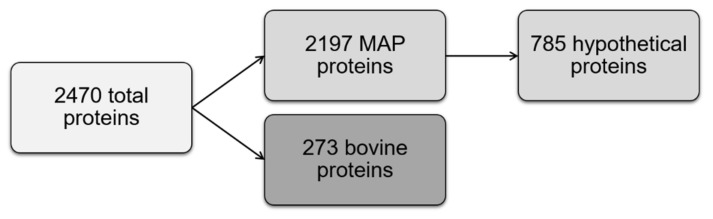
Overview of identified proteins. Of the total of 2470 proteins identified, 237 were classified as bovine and 2197 were linked to MAP. Furthermore, 785 of identified MAP proteins were previously not described on a proteomic level and therefore thus far classified as hypothetical proteins.

To analyze if Mycobacterium avium ssp. paratuberculosis (MAP) changes its protein expression through incubation in milk, we performed a differential proteomics experiment using quantitative liquid chromatography–tandem mass spectrometry (LC–MS/MS) analysis of MAP incubated in Middlebrook 7H9 (M7H9) standard cell culture medium and milk at 37 °C and at 4 °C. Milk at 37 °C simulated the condition in cow’s udder, and milk at 4 °C the simulated condition in bulk tank. To eliminate the highly abundant milk proteins prior to measurement, we refiltrated the MAP samples after incubation with a novel method. A total of 2470 proteins were identified (Table S1), divided into 2197 MAP proteins and 273 bovine proteins. Of these 2197 MAP proteins, 785 (36%) were classified as “hypothetical proteins” in the gene bank entries (Figure 1). For further analyses, we searched and added the gene IDs for these candidates (Table S2).

### 2.2. Highly Differential MAP Proteomes after Incubation in Standard Medium and Milk

**Figure 2:**
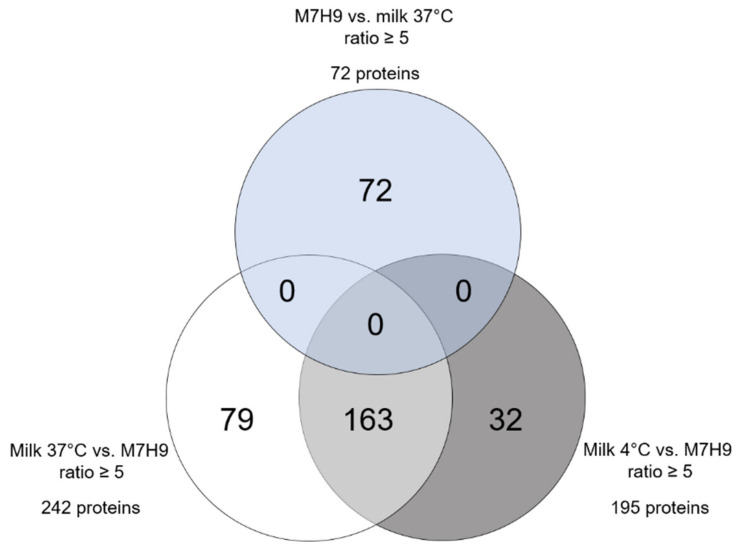
Differentially abundant (ratio ≥ 5) MAP proteins between milk and M7H9 medium. After incubation in milk at 37 °C (white circle) and 4 °C (gray circle), a total of 274 MAP proteins were at least fivefold higher in abundance compared to incubation in M7H9 broth. Most of the proteins (163, light gray) were enriched in milk at both temperatures, while 79 showed increased abundance only in milk at 37 °C (white) and 32 proteins only in milk at 4 °C (dark gray). In contrast, 72 proteins were at least fivefold higher abundant in M7H9 broth compared to milk at 37 °C (blue).

A large difference between proteomes of MAP incubated in milk and in M7H9 became evident, whereas the temperature of the milk exerted a less strong effect on differential protein expression (Figure 2). In total, 274 MAP proteins were fivefold higher abundant in MAP incubated in milk at both temperatures compared to MAP from M7H9 broth (Figure 2, white and gray circles). Therein, 195 proteins were higher abundant in MAP from milk at 4 °C compared to the MAP proteome in M7H9 medium (Figure 2, gray circle). A similar difference was detected between the proteome of MAP incubated in milk at 37 °C and MAP in medium, revealing 242 proteins that were at least five times higher in their abundance in milk at 37 °C (Figure 2, white circle). Out of those proteins, the majority (163 proteins; Figure 2, light gray) were higher in abundance in both milk samples, whereas 32 proteins were exclusively enriched in milk at 4 °C (Figure 2, dark gray) and 79 proteins only in milk at 37 °C (Figure 2, white). In contrast to the 274 higher abundant MAP proteins in milk, with 91 proteins, only one-third as many proteins showed fivefold increased abundance after incubation in M7H9 broth compared to milk at both temperatures. In more detail, 72 proteins were higher abundant in medium compared to milk at 37 °C (Figure 2, light blue) and 54 proteins compared to milk at 4 °C (data not shown).

### 2.3. Functional Affiliation of Differential MAP Proteins

**Figure 3:**
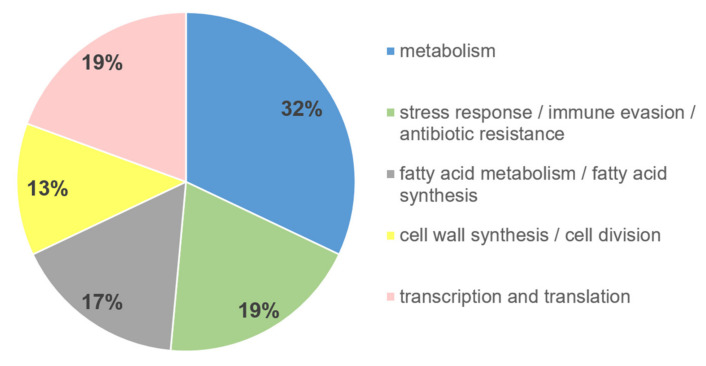
Functional affiliation of higher abundant MAP proteins in milk. The proteins fivefold higher in abundance in both milk at 37 °C and 4 °C compared to M7H9 broth can be functionally assigned to 32% metabolism (blue), 19% transcription and translation (pink), 19% stress response/immune evasion/antibiotic resistance (green), 17% each fatty acid metabolism/fatty acid synthesis (gray), and 13% cell wall synthesis/cell division (yellow).

To further analyze the functional impact of the differentially (ratio ≥ 5) higher abundant MAP proteins in milk compared to M7H9 medium, we assigned the proteins to their function. The analysis revealed that 32% of the proteins were assigned to the function metabolism (Figure 3, blue), 19% to each stress response/immune evasion/antibiotic resistance (Figure 3, green) and to transcription/translation (Figure 3, pink), 17% to fatty acid metabolism/synthesis (Figure 3, gray), and 13% to cell wall synthesis/cell division (Figure 3, yellow).

Table 1 provides an overview of the top three proteins from each functional affiliation.

### 2.4. Stably Expressed Proteins as Possible Targets for Detection Methods

In order to find a possible target molecule for MAP detection in food, we screened the respective proteomes for molecules, which were stably expressed in all three environments tested. Thus, a group of 35 proteins that were highly abundant (>20 peptide counts) on one hand and quite equally expressed (ratio ≤ 2) in all three conditions on the other hand were identified (Table 2).

## 3. Discussion

MAP infections in ruminants reduce milk production and lead to premature removal as well as reduced proceeds from subsequent sales of respective MAP-infected animals. MAP belong to the slow-growing non-tuberculous mycobacteria (NTM) species and can cause opportunistic infections in animals and humans [34,35]. However, MAP is also described as a food-borne pathogen [6], thought to be mainly transmitted via milk and dairy products [7,14].

Since bacteria generally sense environmental changes and respond with various mechanisms [23], we were interested in changes in the MAP proteome after incubation in milk. With our study, we provide a comprehensive characterization of MAP proteome cultured in short-time high-temperature, defatted, and casein partially removed milk. The aim of this pretreatment procedure was to remove bacteria in order to avoid interference and to allow unambiguous assigning of proteins. Fat had to be removed because it impaired the filtration process. In milk of most species, approximately 97% of the fat is present in fat globules, which consist of triglycerides surrounded by a membrane [36]. Fat globules, but not other minor lipids such as cholesterol, phospholipids, diacylglycerol, and free fatty acids, were removed by the pretreatment, thus resembling conditions comparable to skimmed milk. The fact that caseins were detectable in the mass spectrometry data set (Table S1) indicates that a significant proportion of casein micelles were able to pass the filter. Thus, the experimental outcomes should also not have been influenced by lack of casein. According to the literature, heat-treated skimmed milk offers favorable conditions for metabolic activity of lactic acid bacteria [37]. Therefore, we did not expect major differences in the conditions for bacterial metabolism between our pretreated milk and milk in field conditions. Using label-free LC–MS/MS, we investigated changes in the MAP proteome after incubation in milk at 37 °C and 4 °C in comparison to MAP incubated in M7H9 broth at 37 °C in this study. Milk at 37 °C should simulate the condition in the bovine udder and milk at the 4 °C condition in the milk bulk tank, while M7H9 broth served as comparison as a standard cell culture medium for MAP. In this approach, a total of 2470 proteins were identified (Table S1). Therein, 273 proteins were from bovine origin and 2197 were MAP proteins, from which 785 were classified as “hypothetical proteins” in the gene bank entries (Figure 1). Hypothetical proteins are predicted to be expressed from an open reading frame, but there is no experimental evidence of translation into proteins and the associated function [38]. The expression of the 785 hypothetical proteins in MAP in vivo was thus confirmed by our study for the first time and underlines the analytical depth of the mass spectrometric analysis. Hypothetical proteins can be crucial for the pathogen survival and the severity of the associated disease in the host [39,40]. For many microorganisms, amongst others for *Mycobacterium tuberculosis* [41], hypothetical proteins were identified to have great importance, as they could serve as potential drug targets or vaccine candidates [42,43]. Thus, the high number of hypothetical proteins identified in our study also holds high potential as new candidates for detection methods of MAP and therefore adds important novel information about this genus on the protein level. Looking at the differentially expressed (ratio ≥ 5) MAP proteins between the three different approaches, we found a clear quantitative difference between the MAP proteome incubated in milk and in M7H9 medium. In total, 274 MAP proteins were at least fivefold higher in abundance in milk at both temperatures compared to M7H9 broth (Figure 2, white and gray circles), whereas after incubation in M7H9 broth, only one-third as many proteins (91 proteins) showed at least fivefold increase of abundance compared to milk at both temperatures. Interestingly, the temperature of the milk had less influence on the proteome changes (Figure 2). Thus, from the 274 differentially higher expressed MAP proteins in milk, with 163 proteins, the major part was higher abundant in both milk samples, irrespective of the temperature (Figure 2, light gray).

Since we were particularly interested in how MAP adapts to the milk environment, we looked more closely at the functions of the differentially higher abundant MAP proteins in milk. The major part of those proteins was assigned to the function cell metabolism (Figure 3, blue, 32%). Deoxyuridine 5′-triphosphate nucleotidohydrolase (Dut) was exclusively detected in MAP incubated in milk, but not in broth. The intact Dut protein is essential in NTM mycobacteria and plays an exclusive role for biosynthesis and growth [44].

In *Saccharomyces cerevisiae*, the inactivation of Dut was lethal because of genome instability [45]. We do not know why MAP upregulated Dut when cultivated in milk, but the concomitant higher abundance of key metabolic enzymes NAD-dependent aldehyde dehydrogenase AldA_1 and the uridylate kinase PyrH point to a stimulation of metabolic pathways.

In mammals, Alda_1 activates mitochondrial aldehyde dehydrogenase 2 (ALDH2), which is generally known for its protective functions [46]. Thus, ALDH2 attenuates oxidative damage of various cell types in humans, including cardiac, lung, and brain endothelial cells and hepatic cells, and it also reduces damage in various organs in animal models [46,47,48,49,50,51,52]. Inhibition of oxidative stress responses in hemorrhagic shock of alveolar damage and of immune cell infiltration were also observed through increased ALDH2 activity via AldA_1 in animal models [50,53]. Taken together, these data could point to stress for MAP in milk environment, resulting in upregulation of AldA_1 as a means to protect themselves against antimicrobial components of milk [24,25]. It is likely that the increased AldA_1 expression in MAP results in attenuating the immune responses. Interestingly, AldA_1 was found in high abundance in tylosin-resistant *S. xylosus* compared with tylosin-sensitive strains [54]. This relationship between increased AldA_1 expression in other bacteria and antibiotic resistance is also of special interest in the context of increased AldA_1 expression of MAP in milk. Another differentially regulated MAP protein allocated to the function metabolism was uridylate kinase (PyrH; Table 1; ratio milk 4 °C/M7H9: 21.2; ratio milk 37 °C/M7H9: 8.8), which catalyzes the phosphorylation of UMP to UDP, further serving as a precursor in the pyrimidine biosynthetic pathway [55]. Kim et al. found the PyrH gene expressed in vivo in *Vibrio vulnificus* during infection in humans [56]. They showed that PyrH inactivation reduced virulence of the bacteria [56]. Further research of this group confirmed an essential role for PyrH for the in vivo survival and growth of *Vibrio vulnificus* [57]. A study comparing the available bacterial genomes revealed that the PyrH gene is highly conserved between bacteria with no counterpart in eukaryotes [55]. In this context, the distinct higher expression of PyrH in MAP after incubation in milk also points to upregulation of immune evasion responses by MAP.

Another 19% of the differentially higher abundant MAP proteins in milk were assigned to the functional affiliations stress response/immune evasion/antibiotic resistance. The top three candidates were RecA (Table 1; ratio milk 4 °C/M7H9 and milk 37 °C/M7H9: infinite), RelA (Table 1; ratio milk 4 °C/M7H9: 313.2; ratio milk 37 °C/M7H9: 8371.5), and SecE (Table 1; ratio milk 4 °C/M7H9: 26.5; ratio milk 37 °C/M7H9: 25.4). RecA is important for DNA damage repair [58]. SecE translocates proteins across the plasma membrane [59]. RelA plays a central role in the ability of MAP to establish a persistent infection [60]. Deletion of RelA led to immune elimination of the mutant MAP [60].

Candidates enriched from transcription/translation pathway, DnAE, as well as XthA and SigG, belong to the stressome of MAP [61]. XthA acts on damaged DNA in bacterial base excision repair [62]. SigG is upregulated by DNA-damaging agents and by macrophage infection [63].

Top enriched candidates from fatty acid metabolism and synthesis were FadD5 (Table 1; ratio milk 4 °C/M7H9: 122.9; milk 37 °C/M7H9: 103.1), which may serve to recycle mycolic acids for the long-term survival of the tubercle bacilli [64], MmpL4_1 and LipN. MmpL4_1 (Table 1; ratio milk 4 °C/M7H9 and milk 37 °C/M7H9: infinite) is important for virulence of mycobacteria through secretion of specific lipids and participates in export of mycobacterium cell wall components to the bacterial surface [65,66]. LipN (Table 1; ratio milk 4 °C/M7H9: 6.0; milk 37 °C/M7H9: 5.9) is a fatty acid degradation lipase/esterase that was shown to be significantly upregulated in MAP shed in cow feces [61].

CrgA (Table 1; ratio milk 4 °C/M7H9: 14.1; milk 37 °C/M7H9: 18.5), MmpL11 (Table 1; ratio milk 4 °C/M7H9: 12.2; milk 37 °C/M7H9: 13.5), and YidC (Table 1; ratio milk 4 °C/M7H9: 6.7; milk 37 °C/M7H9: 7.4) were the top enriched candidates from cell wall synthesis/cell division. CrgA is related to peptidoglycan biosynthesis and cell shape maintenance [67]. MmpL11 is a conserved transporter of mycolic acid-containing lipids, which was shown to be important for biofilm formation in M. tuberculosis and M. smegmatis, as well as in non-replicating persistence in M. tuberculosis [68]. The membrane protein insertase YidC is required for the insertion and proper folding and complex formation of integral membrane proteins into the membrane of many bacteria [69].

Our analyses demonstrated significant changes in MAP proteome after cultivation in milk for 48 h. Higher abundant proteins have functions in protection and repair of MAP and point to a stressome reaction in milk. Milk was apparently sensed as a hostile environment and therefore induced respective counteractions by MAP.

Significant proteome changes after cultivation in milk compared to cultivation in a standard medium have already been shown for *Escherichia coli*, *Lactococcus lactis*, and *Streptococcus thermophilus* [24,26,27]. Our study confirms similar responses of MAP in milk and provides in-depth information about respective stress response of MAP in milk. Respective candidate proteins and pathways deserve further investigations to better understand interactions of MAP with milk. The next experiments should clarify if and how MAP survive in this environment over longer periods of time, since the survival of MAP in dairy products is unwanted. The better understanding of factors that prevent persistence of MAP could aid to inhibit resistance strategies of MAP. Perhaps subsequent analyses could also explain environment-dependent variations such as cell wall-deficient forms of MAP that were repeatedly isolated from human intestines [28,29,30].

Another focus of our study was on identifying possible candidate proteins for new detection methods for MAP, as there is still a lack of a rapid and sensitive detection method for MAP [70]. The gold standard for MAP detection is currently cultivation on agar [71]. This method has the advantage of detecting only live MAP, but takes weeks to produce a result, which is not suitable for detection in food [72]. Detection by PCR provides rapid results and is very sensitive, but it cannot distinguish between live and dead MAP [71,73]. In addition, false positive identifications may occur due to identification of non-MAP mycobacteria [74]. Thus, there is a need for rapid, sensitive, and highly specific detection of MAP, both in livestock and food. The solution could be an antibody-based assay. The candidate protein sought for this should ideally meet the following conditions: highly abundant expression in all conditions tested, MAP-specific epitope present, and being located at the bacterial surface. For this purpose, we first evaluated the most abundant proteins from the data set that were uniformly highly expressed between all conditions (ratio ≤ 2). This analysis yielded 35 proteins (Table 2), which will now be further investigated to identify MAP-specific conserved candidate proteins. In silico, all proteins with at least two unique peptides can be translated into DNA sequences and aligned with all contained total genomes and sequences of the Mycobacterium avium complex (MAC) in order to identify sequences that differ significantly from those of other MAC representatives unequal to MAP. In addition, the candidate protein should preferably be expressed at the MAP surface, as this would greatly facilitate the detection of this bacterium.

## 4. Materials and Methods

### 4.1. Bacteria

*Mycobacterium avium* subsp. *paratuberculosis* (MAP) strain DSM 44133 was obtained from the German Collection of Microorganisms and Cell Cultures (DSMZ, Braunschweig, Germany). MAP were cultivated for four weeks on Herrold’s egg yolk agar (HEYM) (BD Biosciences, Heidelberg, Germany).

### 4.2. Preparation of Milk

For defatting, fresh milk (1.5% fat, short time high temperature (72 °C, 20 s)) was centrifuged (20 min, 2000× *g*, 4 °C), and the resulting fat layer was removed. After filtration with a paper filter, milk was additionally filtered with 0.45 µm followed by 0.2 µm filters, partially removing casein. For contamination, control milk was incubated at 37 °C for 24 h and streaked out on blood agar, which was incubated at 37 °C for another 48 h. No microbial growth was detected.

### 4.3. Incubation of MAP in Liquids

MAP were harvested from HEYM agar by careful flushing with PBS. After washing three times with PBS (10 min, 16,000× *g*), 2 × 10^9^ cfu/mL MAP were inoculated into two aliquots of the prepared milk and in Middlebrook 7H9 (M7H9; Sigma-Aldrich, Taufkirchen, Germany) medium as liquid control medium. One preparation of the artificially contaminated milk and the control medium preparation were incubated at 37 °C, and the other aliquot of milk at 4 °C for 48 h. Milk at 37 °C should simulate the condition in the bovine udder and the milk at the 4 °C condition in the milk bulk tank, while M7H9 broth served as comparison as a standard cell culture medium for MAP.

### 4.4. Harvesting and Refiltration of MAP

All three assays were shortly warmed up to 38 °C in order to perform the following refiltration with as little pressure as possible. For refiltration of MAP, all preparations were first filtrated through 0.45 µm filters, followed by washing the filters two times with PBS. To obtain the MAP collected in the filters, we backflushed the corresponding filters (in opposite direction) with PBS. Extracted MAP-PBS suspension was centrifuged (30 min, 2500× *g*) and remaining supernatants were again centrifuged (10 min, 16,000× *g*) to make sure that all MAP were sedimented. Finally, related MAP pellets were pooled and washed three times with PBS (10 min, 16,000× *g*).

### 4.5. Lysis of MAP Samples

MAP pellets were resuspended in lysis buffer (1% Nonidet P-40, 10 mM NaCl, 10 mM Tris-HCl (pH 7.6)). For additional breakup of the robust MAP cell wall, samples were treated with silica beads (0.1 mm; 40 mg beads/100 µL lysate) in Ribolyser (Hybaid, Teddington, UK; level 6, 3 × 30 s). Heating (74 °C, 5 min) followed by ultrasonic treatment (6 × 30 s on ice) of samples gave maximum split-up.

### 4.6. Sample Preparation for LC–MS/MS Mass Spectrometry

A total of 10 µg total protein was digested with LysC and trypsin by a modified filter-aided sample preparation (FASP) [75] as follows. To every lysate, 1 µL 1 M dithiothreitol was added and incubated for 30 min at 60 °C. After cooling down, samples were diluted 1:3 in urea (UA)-buffer (8 M urea and 0.1 M Tris-HCl (pH 8.5) diluted in HPLC-grade water) and incubated with 10 µL of 300 mM iodoacetamide for 30 min at room temperature in the dark. After addition of 2 µL 1 M dithiothreitol, samples were transferred to 30 kDa cut-off centrifuge filters (Sartorius, Göttingen, Germany) and centrifuged (15 min, 14,000× *g*), followed by three washing steps with each 200 µL UA-buffer and 100 µL ammoniumbicarbonate (ABC) buffer (50 mM diluted in HPLC-grade water). Afterwards, the proteins were subjected to proteolysis with 1 µg Lys C (Lysyl Endopeptidase C) in 40 µL ABC buffer at 37 °C overnight. After centrifugation (15 min, 15,000× *g*) over new tubes and renewed washing with 20 µL ABC buffer, collected Lys-C fractions were acidified with 0.5% trifluoroacetic acid to pH 2 and frozen at −20 °C. Filter were subsequently incubated with 2 µg trypsin in 50 µL of ABC buffer at 37 °C overnight. Peptides of tryptic digestion were collected by centrifugation (15 min, 15,000× *g*) and additional washing with 20 µL ABC buffer containing 5% acetonitrile, and were also finally acidified with 0.5% trifluoroacetic acid to pH 2.

### 4.7. Mass Spectrometry

Acidified eluted peptides were analyzed in the data-dependent mode on a Q Exactive HF mass spectrometer (Thermo Fisher Scientific, Bremen, Germany) online coupled to a UItimate 3000 RSLC nano-HPLC (Dionex, Idstein, Germany). Samples were automatically injected and loaded onto the C18 trap column, and after 5 min eluted and separated on the C18 analytical column (75 µm ID × 15 cm, Acclaim PepMAP 100 C18. 100Å/size, LC Packings, Thermo Fisher Scientific, Bremen, Germany) by a 90 min non-linear acetonitrile gradient at a flow rate of 250 nL/min. MS spectra were recorded at a resolution of 60,000, and after each MS1 cycle, the 10 most abundant peptide ions were selected for fragmentation.

### 4.8. Protein Identification and Label-Free Quantification

Acquired MS spectra were imported into Progenesis software (version 2.5, Nonlinear Dynamics, Waters, Oslo, Norway) and analyzed as previously described [76,77]. After alignment, peak picking, exclusion of features with charge state of 1 and >7 and normalization, spectra were exported as Mascot Generic files and searched against a database containing all entries of *Mycobacterium avium* subspecies *paratuberculosis* from NCBI Protein database combined with the Ensembl bovine database (version 80) with Mascot (Matrix Science, Version 2.5.1). Search parameters used were 10 ppm peptide mass tolerance, 20 mmu fragment mass tolerance, one missed cleavage allowed, carbamidomethylation set as fixed modification, and methionine oxidation and deamidation of asparagine and glutamine as variable modifications. Mascot integrated decoy database search was set to a false discovery rate (FDR) of 1% when searching was performed on the concatenated mgf files with a percolator ion score cut-off of 13 and an appropriate significance threshold p. Peptide assignment was reimported to Progenesis Software. All unique peptides allocated to a protein were considered for quantification.

### 4.9. Data Analysis

Proteins with a ratio of at least fivefold in normalized abundance between samples were defined as differentially expressed. Venn diagram was calculated with Venny 2.1 open source tool (https://bioinfogp.cnb.csic.es/tools/venny/ (accessed on 14 May 2021)). To determine the function of the differentially expressed proteins (ratio ≥ 5) between milk at both temperatures and M7H9 broth, we first manually searched the respective proteins by accession number at Ensembl Bacteria (release 51). The functions of the domains indicated there were subsequently determined via InterPro (release 85.0; http://www.ebi.ac.uk/interpro/ (accessed on 20 May 2021)) and finally illustrated in a pie chart.

## 5. Conclusions

MAP proteomes from MAP incubated in milk show distinct modifications to this environment. The majority of changed proteins point to a stress response of MAP and a subsequent reaction to cope with nutrient changes and the hostile environment milk, leading to DNA damage and other impairments in MAP. However, there were also stably expressed proteins detected in this study that could be suited as targets for MAP detection.

## Figures and Tables

**Table 1:** Selection of differentially higher abundant MAP proteins in milk assigned to functional affiliation.

Protein ID	Accession	Peptide Count	Ratio			Functional Affiliation
			Milk 4 °C/M7H9	Milk 37 °C/M7H9	Milk 37 °C/Milk 4 °C	
Dut	AAS05131	1	infinite	infinite	0.5	metabolism
AldA_1	AAS02919	1	75.9	23.4	0.3	
PyrH	AAS05263	2	21.2	8.8	0.4	
DnaE1	AAS03574	2	939.7	1365.0	1.5	transcription and translation
SigG	AAS06171	1	23.7	42.9	1.8	
XthA	AAS06466	1	17.1	16.6	1.0	
CrgA	AAS02330	4	14.1	18.5	1.3	cell wall synthesis/cell division
MmpL11	AAS06187	3	12.2	13.5	1.1	
YidC	AAS06897	4	6.7	7.4	1.1	
MmpL4_1	AAS02393	1	infinite	infinite	1.6	fatty acid metabolism/fatty acid synthesis
FadD5	AAS06151	2	122.9	103.1	0.8	
LipN	AAS05554	2	6.0	5.9	1.0	
RecA	AAS05165	1	infinite	infinite	0.7	stress response/immune evasion/antibiotic resistance
RelA	AAS03364	3	313.2	8371.5	26.7	
SecE	AAS06660	3	26.5	25.4	1.0	

**Table 2:** Highly abundant proteins, which were similarly expressed in all different test conditions (milk 37 °C, milk 4 °C, M7H9 broth).

	Protein ID	Accession	Peptide Count	Ratio		
				Milk 4 °C/M7H9	Milk 37 °C/M7H9	Milk 4 °C/Milk 37 °C
1	RpoC	AAS06681	55	1.0	1.4	0.7
2	Fas	AAS04649	41	1.4	0.7	2.0
3	Kgd	AAS04853	41	0.8	1.1	0.7
4	RpoB	AAS06680	41	0.6	0.8	0.8
5	MetE	AAS04978	39	0.7	0.9	0.8
6	AceAb	AAS03960	34	0.9	1.3	0.7
7	NAD-glutamate dehydrogenase	ETB03859	33	1.5	1.3	1.1
8	Catalase/hydroperoxidase HPI(I)	ETB03832	32	1.2	0.6	1.8
9	Aconitate hydratase	ETB10875	30	1.3	1.3	1.0
10	GyrA	AAS02323	29	0.9	1.0	0.9
11	Glutamate synthase	ETB00293	29	1.8	1.5	1.2
12	Rne	AAS04584	28	0.8	1.0	0.8
13	MmpL3	AAS06191	28	0.8	0.8	0.9
14	3-Hydroxyacyl-CoA dehydrogenase	ETB02135	28	1.2	1.1	1.2
15	IleS	AAS03563	27	0.9	0.9	1.1
16	MAP_3698c	AAS06248	27	1.0	1.2	0.8
17	Long-chain fatty acid-CoA ligase	ETB02782	27	0.5	0.6	0.9
18	CtpI	AAS06048	26	1.4	1.1	1.3
19	PckA	AAS06196	26	1.0	0.5	1.9
20	ClpC	AAS02778	25	0.6	0.9	0.7
21	Transketolase	ETB00031	25	1.0	0.8	1.2
22	DNA topoisomerase I	ETB01242	25	1.5	1.7	0.8
23	ABC transporter ATP-binding protein	ETB03858	25	0.8	0.9	1.0
24	PonA_1	AAS02381	24	0.6	0.9	0.6
25	PolA	AAS03639	24	0.7	0.8	0.8
26	ValS	AAS04588	24	0.7	0.8	0.8
27	Rho	AAS04781	24	1.0	1.2	0.8
28	SahH	AAS05912	24	1.8	2.0	0.9
29	Pyruvate dehydrogenase E1	ETB03102	24	0.5	0.8	0.7
30	ATP synthase subunit beta	ETB04389	24	1.4	1.1	1.3
31	Pca	AAS02611	23	1.6	1.3	1.2
32	GltA2	AAS03146	23	0.9	0.7	1.2
33	GlnE	AAS04282	22	1.6	1.7	0.9
34	InfB	AAS05224	21	0.6	0.9	0.7
35	MAP_3291c	AAS05841	21	0.7	1.1	0.7

## Data Availability

The data presented in this study are available in Table S1: All identified proteins. The mass spectrometry proteomics data have been deposited to the ProteomeXchange Consortium via the PRIDE [78] partner repository with the dataset identifier PXD027444.

## Supplementary Materials

The following data are available online at https://www.mdpi.com/article/10.3390/metabo11080549/s1, Table S1: All identified proteins, Table S2: Hypothetical MAP proteins.
